# Metagenomic Guilt by Association: An Operonic Perspective

**DOI:** 10.1371/journal.pone.0071484

**Published:** 2013-08-06

**Authors:** Gregory Vey

**Affiliations:** Department of Biology, University of Waterloo, Waterloo, Ontario, Canada; J. Craig Venter Institute United States of America

## Abstract

Next-generation sequencing projects continue to drive a vast accumulation of metagenomic sequence data. Given the growth rate of this data, automated approaches to functional annotation are indispensable and a cornerstone heuristic of many computational protocols is the concept of guilt by association. The guilt by association paradigm has been heavily exploited by genomic context methods that offer functional predictions that are complementary to homology-based annotations, thereby offering a means to extend functional annotation. In particular, operon methods that exploit co-directional intergenic distances can provide homology-free functional annotation through the transfer of functions among co-operonic genes, under the assumption that guilt by association is indeed applicable. Although guilt by association is a well-accepted annotative device, its applicability to metagenomic functional annotation has not been definitively demonstrated. Here a large-scale assessment of metagenomic guilt by association is undertaken where functional associations are predicted on the basis of co-directional intergenic distances. Specifically, functional annotations are compared within pairs of adjacent co-directional genes, as well as operons of various lengths (i.e. number of member genes), in order to reveal new information about annotative cohesion versus operon length. The results suggests that co-directional gene pairs offer reduced confidence for metagenomic guilt by association due to difficulty in resolving the existence of functional associations when intergenic distance is the sole predictor of pairwise gene interactions. However, metagenomic operons, particularly those with substantial lengths, appear to be capable of providing a superior basis for metagenomic guilt by association due to increased annotative stability. The need for improved recognition of metagenomic operons is discussed, as well as the limitations of the present work.

## Introduction

The ongoing prevalence of next-generation sequencing projects continues to drive a vast accumulation of metagenomic sequence data. As of 2011, the Sequence Read Archive [1] exceeded 100 Terabases of open-access reads produced by next-generation sequencing efforts, with metagenomic sequences accounting for an 11% share of all bases [2]. Moreover, this trend seems unlikely to subside with the imminent arrival of faster and less expensive sequencing technologies [3]. Given the growth rate of metagenomic data, automated approaches to functional annotation are indispensable. A cornerstone heuristic of many computational protocols for functional annotation is the concept of guilt by association (GBA) which asserts that genes that are associated by way of protein interactions or expression patterns are more likely to share a function [4]. As a result, GBA has been heavily exploited by both gene co-expression research [5]–[8] and studies involving genomic context methods [9]–[12].

Genomic context methods are of particular interest to functional annotation efforts because they offer functional predictions that are complementary to homology-based annotations [13], [14], thereby offering a means to extend the total proportion of annotation. However, methods involving phylogenetic profiles [15], [16], conserved gene orders [17], [18], and gene fusions [19], [20] still require determinations of orthology that are not possible when using metagenomic sequence fragments because they are not equivalent to discrete and intact genomes [21]. In contrast, operon methods that exploit co-directional intergenic distances offer context-based predictions and have been used previously to determine metagenomic functional associations [21]–[23]. Therefore, metagenomic operons can provide homology-free functional annotation through the transfer of functions among co-operonic genes, assuming that GBA is indeed applicable to metagenomic scenarios.

Although GBA remains a well-accepted annotative device, its merit has met with both early [24] and ongoing opposition [25]. Moreover, its specific applicability to metagenomic functional annotation has not been definitively demonstrated. In the present work a large-scale assessment of metagenomic GBA is undertaken where associations are predicted on the basis of co-directional intergenic distances. Specifically, functional annotations are compared within pairs of adjacent co-directional genes, as well as operons of various lengths (i.e. number of member genes), in order to reveal new information about annotative cohesion versus operon length. The effects of multifunction genes are also considered.

## Methods

Both metagenomic and genomic genes were parsed from downloaded raw data and used to derive gene pair data and to construct a database of metagenomic operons. The data were subsequently mined to obtain individual gene pair comparisons, as well as whole operon comparisons. All operations were computationally implemented in Java and run on a Gateway NV59 laptop using an Intel Core i3-330M processor.

### Data Acquisition

The raw metagenomic data consisted of the complete set of public metagenomes available from the Integrated Microbial Genomes with Microbiome Samples (IMG/M) metagenomics database [26] as of February 1^st^ 2012. This included 305 total datasets with the following exceptions: i) 16 datasets were unobtainable due to their file sizes and the timeout policies of the IMG/M; ii) four datasets were removed because they did not contain gene coordinate information necessary for predicting gene interactions. The remaining 285 datasets contained a total of 47,385,410 protein-coding genes distributed across 45,167,094 scaffolds. The specific features of the individual datasets (e.g. ecosystem, scaffold count, gene count, etc.) are provided as Supporting Information (see Table S1). In addition, the first field (Usage Status) lists the IMG/M identifier for each metagenome in combination with the following colour codes: GREEN = downloaded and used in this study; YELLOW = downloaded but removed due to missing coordinate data; RED = unable to obtain due to file sizes and the timeout policies.

Raw genomic data was also acquired for the construction of genomic gene pairs (see below). Specifically, the.ptt file for the *Escherichia coli* strain K-12 substrain MG1655 genome was downloaded from the National Center for Biotechnology Information (NCBI) FTP directory of bacterial genomes [27] on November 15^th^ 2012. This file included coordinate information and functional annotations for 4,146 protein-coding genes.

### Gene Pair Selection

Intergenic distances in base pairs (bp) were recorded for adjacent pairs of protein-coding genes occurring in the same strand within the same metagenomic scaffold or complete genome if the following conditions were met: i) each gene had exactly one COG functional category [28] annotation (the presence or absence of other additional types of functional annotations had no effect on pair selection); ii) each COG functional category annotation was neither [R] (General function prediction only), nor [S] (Function unknown). For the metagenomic data a total of 92,512 gene pairs were obtained from which 720 pairs (<1%) were removed on the basis of influential observations (intergenic distances>500 bp) leaving 91,792 remaining metagenomic gene pairs. The metagenomic gene pair data are provided as Supporting Information (see Dataset S1). For the genomic data a total of 1,834 gene pairs were obtained from which 59 pairs (3%) were removed on the basis of influential observations (intergenic distances>500 bp) leaving 1,775 genomic gene pairs. The genomic gene pair data are also provided as Supporting Information (see Dataset S1).

### Operon Selection

Operons were predicted using a previously published method for identifying metagenomic operons [21], [22]. Specifically, operons were derived from scaffolds containing two or more adjacent genes in the same strand based on intergenic distances (in base pairs) where the likelihood for two genes to be in the same operon given the distance between them was assigned based on the ratio of known genes in operons to known genes in different transcription units found at such distance [29], [30]. A minimum threshold of confidence was selected that is equivalent to a positive predictive value of 0.85 (i.e. 85% of the predictions are expected to consist of true positives), as evaluated against known operons of *Escherichia coli* K12 found in RegulonDB [31]. In addition, operons were retained only if each member gene had at least one COG category annotation and each COG category annotation was neither [R], nor [S]. While it was possible for a member gene to contain more than one COG category annotation (i.e. a multifunction gene), such cases also required that the additional annotations were neither [R], nor [S]. A total of 748,099 operons were constructed of which 115,533 contained one or more multifunction genes. The operon data are provided as Supporting Information (see Dataset S1).

## Results

In order to evaluate the applicability of GBA to metagenomic functional annotation, the connection between annotative cohesion and functional association was examined. The first set of analyses attempted to determine whether genes that share a functional annotation are more likely to be predicted of having a functional association than genes that do not share a functional annotation. This was accomplished by comparing adjacent co-directional metagenomic gene pairs that had either matching or non-matching functional annotations. The second set of analyses used the reverse approach and attempted to measure the extent to which functionally associated genes share their functional annotations. This was accomplished by measuring the ratio of unique annotations to number of member genes in metagenomic operons of varying lengths. For all conducted analyses functional annotation was measured at the level of COG functional category [28] annotations (COGs). COGs (e.g. [C], [D], [E], etc.) were selected because they represent broad functional categorizations with a small lexicon of possible annotation states, thereby permitting straightforward and conclusive determinations of annotative equivalence. Also, for all conducted analyses functional associations were determined based on predictions of gene interactions calculated using co-directional intergenic distances (see Methods) because this approach represents a homology-free protocol that is well suited to metagenomic scenarios [21], [22].

### Gene Pair Analyses

**Figure 1:**
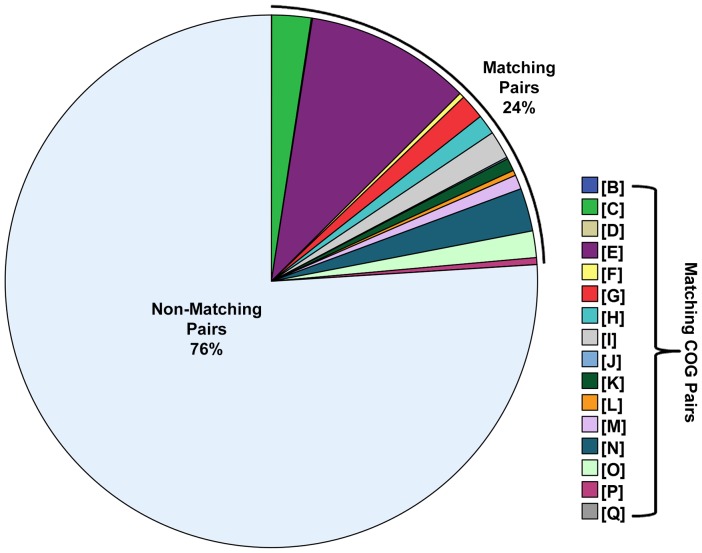
Proportion of Gene Pairs. The relative proportions (%) of gene pairs (Non-Matching versus Matching) are shown with matching gene pairs represented according to their respective types of COG category functional annotations.

**Figure 2:**
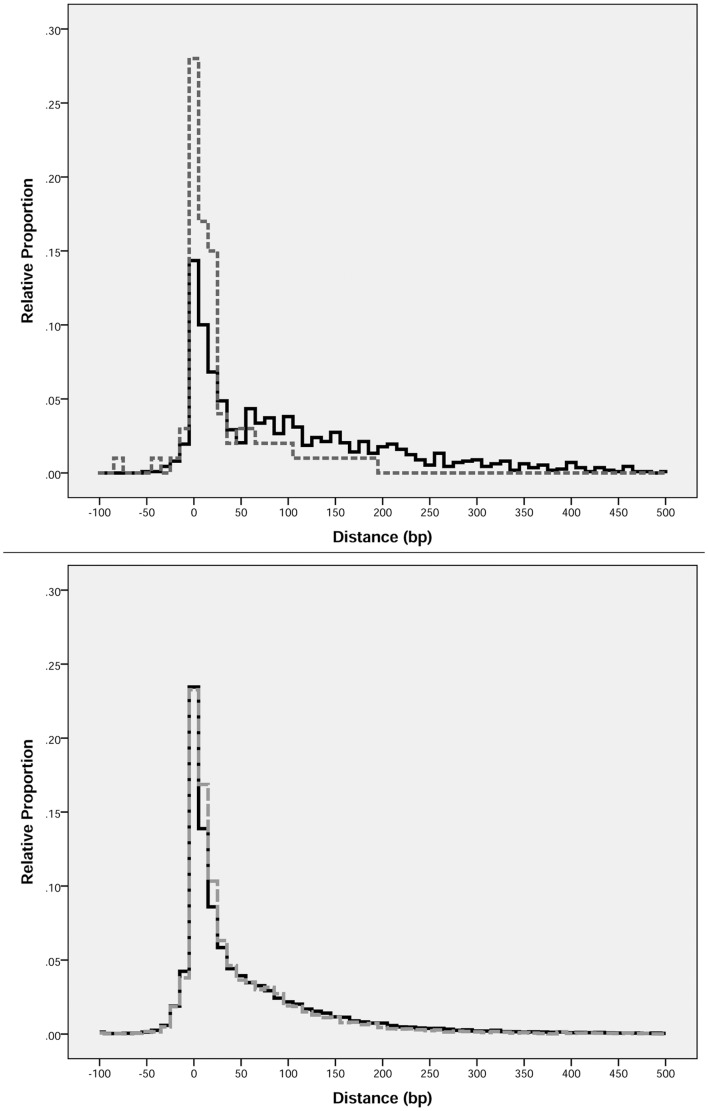
Distribution of Gene Pair Intergenic Distances. The relative proportions (%) of intergenic distances between members of respective gene pairs are shown in 10 base pair (bp) windows for Non-Matching versus Matching pairs. The upper panel shows the distribution of intergenic distances for the *Escherichia coli* K12 MG1655 genome as a reference comparison where Non-Matching pairs are shown in solid black and Matching pairs are shown in broken grey. The lower panel shows the distribution of intergenic distances for the metagenomic gene pairs where Non-Matching pairs are shown in solid black and Matching pairs are shown in broken grey.

In order to determine if genes that share a functional annotation are more likely to be predicted of having a functional association than genes that do not share a functional annotation, the intergenic distances of 91,792 adjacent co-directional gene pairs were recorded, along with their COGs (see Methods). The data were divided into two categories based on the COGs within each gene pair (Non-matching or Matching) with the goal of observing potential differences in the intergenic distances between the categories that would be indicative of different likelihoods of being functionally associated. Non-matching gene pairs (*N* = 69,761) represented 76% of the total cases, while Matching gene pairs (*N* = 22,031) comprised the remaining 24% (see Figure 1). Intergenic distances were compared with respect to match category and both categories exhibited similar right-skewed non-normal distributions (see Figure 2) where Non-matching gene pairs had *M* = 49.29 bp and *SD* = 81.85 bp, while Matching gene pairs had *M* = 40.40 bp and *SD* = 69.61 bp. Table 1 provides an overview of the descriptive statistics for each match category and includes bootstrapped confidence intervals for each reported statistic.

**Table 1:** Descriptive Statistics by Match Category.

Match Category	Statistic		Bootstrapped Values		
			Std. Error	95% Confidence Interval	
				Lower	Upper
Non-Matching	Mean	49.29	0.32	48.63	49.92
	Median	16.00	0.41	15.00	16.00
	Std. Deviation	81.85	0.44	80.97	82.76
	Skewness	2.12	0.02	2.08	2.16
Matching	Mean	40.40	0.46	39.52	41.34
	Median	13.00	0.39	12.00	13.00
	Std. Deviation	69.61	0.75	68.15	71.05
	Skewness	2.34	0.06	2.20	2.46

For each Match Category (Non-Matching vs. Matching) values are reported for the mean, median, standard deviation, and skewness. Bootstrapped values were obtained using 1,000 samples.

To test whether the recorded intergenic distances exhibited discernible differences in range with respect to match category, two binning experiments were conducted using the minimum description length principle [32] for the discretization of scale variables [33], [34] as implemented in [35]. First, all gene pairs were assigned into one of two categories of functional interaction based on their log-likelihood scores (see Methods): Non-interacting or Interacting. Gene pairs were then binned by their intergenic distances to optimize the distinction between Non-interacting versus Interacting pairs. The resulting four bins exhibited a highly resolved categorization where interacting gene pairs were characterized by intergenic distance ranging from −23 bp to 43 bp (see Table 2). The only exception was a small bin (Bin 3) of ambiguous categorizations at 44 bp and 45 bp but this represented less than 1% of the total data. Next, the procedure was repeated and gene pairs were then binned by their intergenic distances to optimize the distinction between Non-matching versus Matching pairs. In contrast to the previous experiment, the result was five highly ambiguous bins where Bin 5 had the greatest resolution with 84% Non-matching pairs and 16% Matching pairs (see Table 3).

**Table 2:** Optimal Binning of Distances by Interaction Category.

Bin	Bounds		Gene Pairs by Interaction Category				Bin Total
	Lower	Upper	Non-interacting		Interacting		
1	Unbound	−23	1,805	(100%)	1	(0%)	1,806
2	−23	44	0	(0%)	58,632	(100%)	58,632
3	44	46	395	(53%)	354	(47%)	749
4	46	Unbound	30,605	(100%)	0	(0%)	30,605

The intergenic distances (bp) between gene pair members were used to create optimal bins with respect to Interaction Category (Non-interacting vs. Interacting) where each bin is demarcated as lower bound≤distance <upper bound. The number of gene pairs in each bin is listed by interaction category, as well as the category proportion (%) of the bin total.

**Table 3:** Optimal Binning of Distances by Match Category.

Bin	Bounds		Gene Pairs by Match Category				Bin Total
	Lower	Upper	Non-matching		Matching		
1	Unbound	−1	17,841	(78%)	5,047	(22%)	22,888
2	−1	23	20,342	(72%)	7,922	(28%)	28,264
3	23	118	21,151	(76%)	6,625	(24%)	27,776
4	118	193	5,817	(79%)	1,533	(21%)	7,350
5	193	Unbound	4,610	(84%)	904	(16%)	5,514

The intergenic distances (bp) between gene pair members were used to create optimal bins with respect to Match Category (Non-matching vs. Matching) where each bin is demarcated as lower bound≤distance <upper bound. The number of gene pairs in each bin is listed by match category, as well as the category proportion (%) of the bin total.

A logistic regression was carried out to determine the following equation that predicts whether or not the COGs in a given gene pair match (i.e. have a value of 1 instead of 0), based on the intergenic distance between its members: *ln*(Odds of Match) = −1.084–0.002(Distance). For example, given an intergenic distance of zero a gene pair is only 0.34 times as likely to have matching COGs as it is to have non-matching COGs (e.g. Odds of Match = *e*?(−1.084–0)). This value can be converted to a probability, *P*(Match) = Odds of Match/1+Odds of Match = 0.254, that predicts that 25% of gene pairs with an intergenic distance of zero will have matching COGs. The regression equation was subsequently used to attempt a classification of the gene pairs with respect to match and showed an overall success rate of 76%. However, this outcome was the result of classifying all gene pairs as Non-matching, thereby yielding a sensitivity of 0% and a specificity of 100% (see Table 4).

**Table 4:** Match Category Classification Table.

		Predicted		Correct
		Non-Matching	Matching	
**Observed**	**Non-Matching**	69,761	0	100%
	**Matching**	22,031	0	0%
**Overall Percentage**				76%

A regression equation was used to classify gene pairs with respect to Match Category (Non-matching vs. Matching). The resulting counts are listed by match category where row totals equal the observed counts and column totals equal the predicted counts. The proportion (%) of correct predictions is also shown.

### Operon Analyses

**Figure 3:**
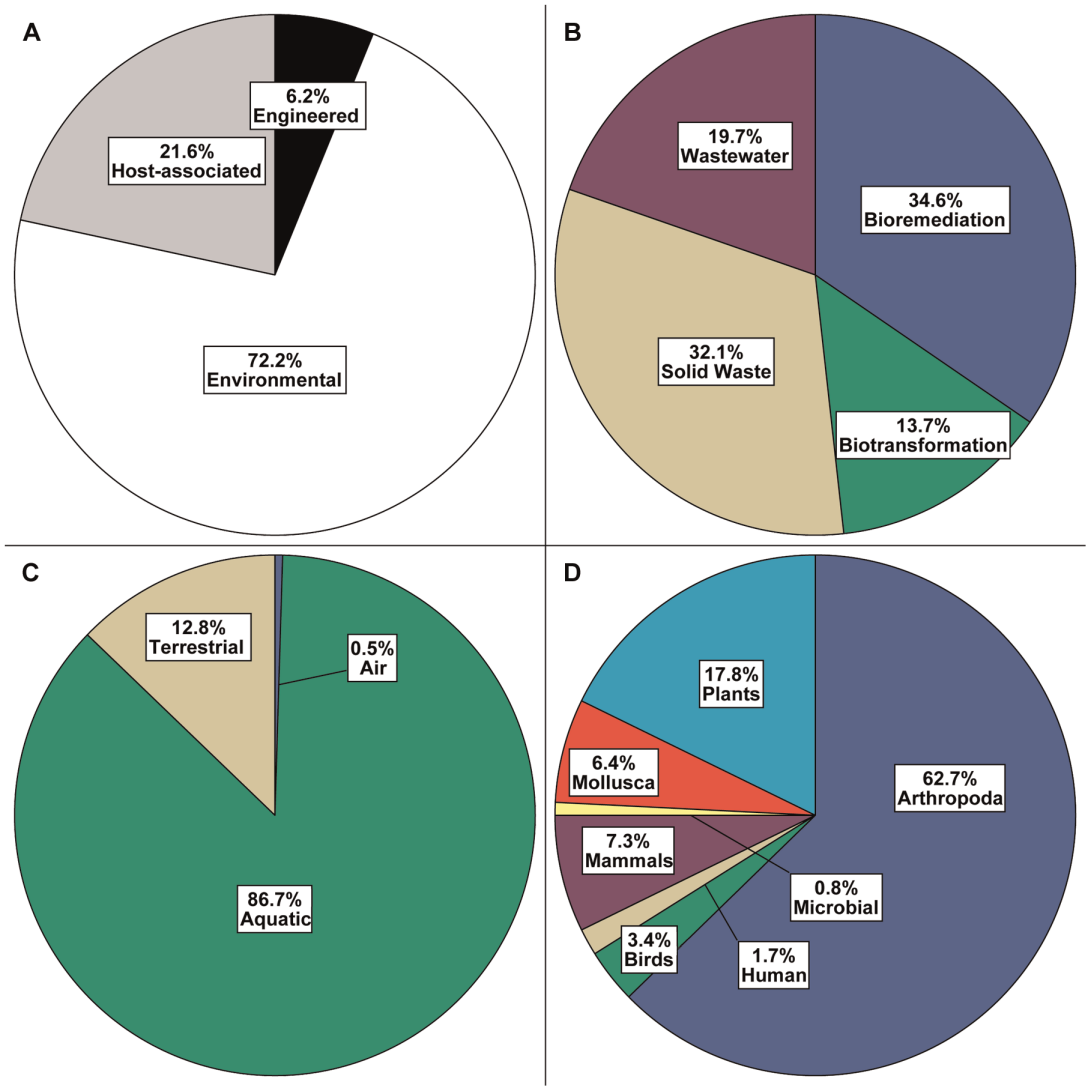
Diversity of Metagenomic Operons. The relative proportions (%) of metagenomic operons are shown with respect to IMG/M Phylum category (A). Within the Engineered phylum, the relative proportions (%) of operons are shown with respect to IMG/M Class category (B). Within the Environmental phylum, the relative proportions (%) of operons are shown with respect to IMG/M Class category (C). Within the Host-associated phylum, the relative proportions (%) of operons are shown with respect to IMG/M Class category (D).

**Figure 4:**
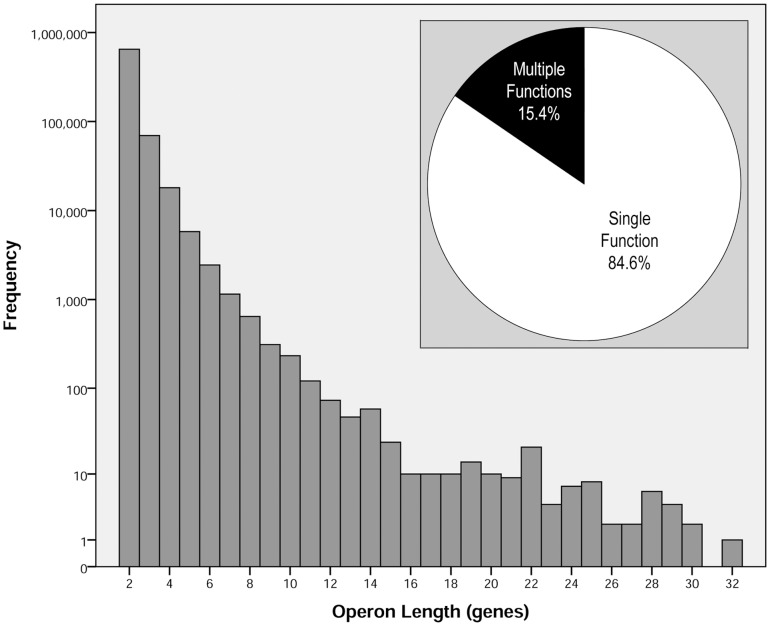
Features of Metagenomic Operons. The main panel shows the distribution of metagenomic operon lengths with respect to frequency of occurrence using a log (base 10) scale. Note, no operons of length 31 were observed. The inset shows the proportion of operons that contain member genes with exactly one COG category annotation (Single Function) versus operons where at least one member gene has more than one COG category annotation (Multiple Functions).

In order to measure the extent to which functionally associated genes share their functional annotations, the annotative cohesion of operons was measured. A large collection of metagenomic operons (*N* = 748,099) was used to ensure source diversity, however the majority (63%) of operons were aquatic (see Figure 3). Operon lengths (i.e. the number of member genes comprising an operon [36], [37]) ranged from 2–32 genes (no operons were observed with a length of 31 genes) with 87% of operons having a length of 2 genes and only 4% of operons having lengths greater than 3 genes (see Figure 4). It should be pointed out that gene pairs from the previous section were equivalent to operons with a length of 2 genes if the pair members were in sufficient proximity to one another (see Methods). In addition, 15% of the operons contained at least one member gene that had more than one COG (see Figure 4).

**Figure 5:**
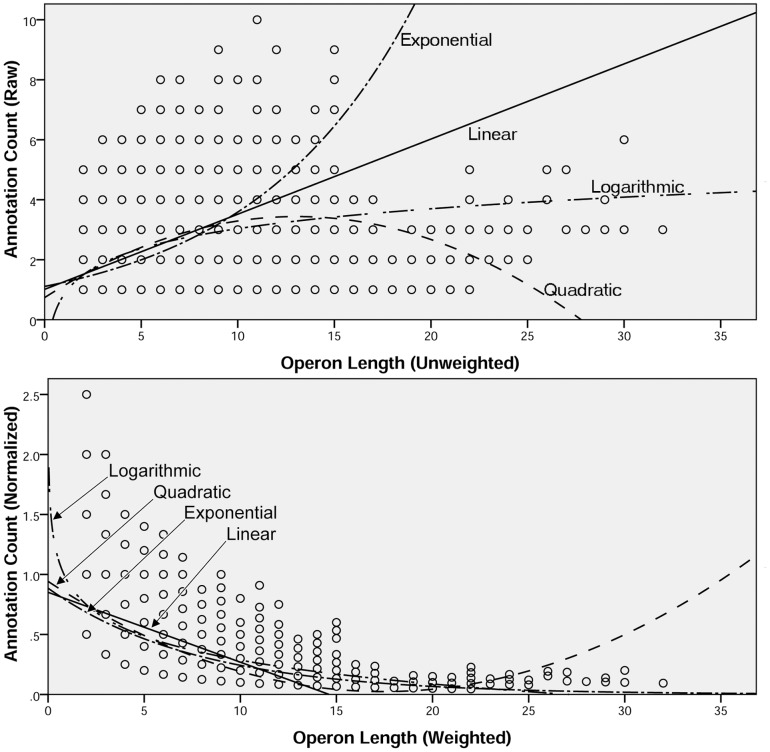
Annotation Count versus Operon Length. Scatterplots are shown for Annotation Count versus Operon Length with four different models superimposed on the observed data. The upper panel displays the models and data for Raw Annotation Count versus Unweighted Operon Length while the lower panel displays the models and data for Normalized Annotation Count versus Weighted Operon Length.

Annotative cohesion was measured by comparing the length of each operon against its unique COG count and several models were fit to the data (see Figure 5). While no clear relationship was found, two key properties were observed: i) annotation count (i.e. unique COG count) grows more slowly than operon length; ii) the relationship for annotation count versus operon length is nonlinear (see Table 5). Because the sample was dominated by short-length operons, the individual cases were reweighted to increase the proportion of longer operon cases. Specifically, each case was weighted by the product of its length and a frequency normalization factor, where the frequency normalization factor was the quotient of the actual number of cases divided by the weighted sum of cases, where the weighted sum of cases was the overall total of each operon length. Also, the annotation count was normalized by dividing the raw annotation count for each operon by its corresponding length. The same models were fitted to the transformed data and the previously observed properties were again evident (see Figure 5). However, this time a clearer relationship (*r^2^* = 0.15, *p*<0.001) was observed in the form of an exponentially decreasing trend where the normalized annotation count decreased rapidly with increasing weighted operon length (see Table 5). Furthermore, to identify effects stemming from the inclusion of operons where at least one member gene had more than one COG (i.e. multifunction genes) the data were split into two sets: single function versus multifunction. The previous models were fitted against each level of function for both raw and transformed versions of the data and again the exponential model was the best fit for the transformed version for both the single function group (*r^2^* = 0.18, *p*<0.001) and the multifunction group (*r^2^* = 0.51, *p*<0.001) (see Table 5). Interestingly each of decomposed datasets had a better value for *r^2^* than the combined dataset suggesting that the relationship for annotation count versus operon length is similar for operons containing only single function genes and operons containing multifunction genes, however the rate of change differs with the multifunction trend being initially higher but decreasing more rapidly until it converges with the single function trend.

**Table 5:** Models for Annotation Count versus Operon Length.

Member Gene Type	Model	Unweighted				Weighted			
		Raw		Normalized		Raw		Normalized	
		*r^2^*	*p*	*r^2^*	*p*	*r^2^*	*p*	*r^2^*	*p*
Both Types Combined (*N* = 748,099)	Linear	0.07	<0.001	0.03	<0.001	0.09	<0.001	0.06	<0.001
	Logarithmic	0.08	<0.001	0.04	<0.001	0.12	<0.001	0.08	<0.001
	Quadratic	0.08	<0.001	0.04	<0.001	0.12	<0.001	0.08	<0.001
	Exponential	0.04	<0.001	0.07	<0.001	0.06	<0.001	0.15	<0.001
Single Function Genes Only (*N* = 632,566)	Linear	0.06	<0.001	0.04	<0.001	0.08	<0.001	0.07	<0.001
	Logarithmic	0.08	<0.001	0.04	<0.001	0.12	<0.001	0.09	<0.001
	Quadratic	0.07	<0.001	0.04	<0.001	0.11	<0.001	0.09	<0.001
	Exponential	0.04	<0.001	0.09	<0.001	0.05	<0.001	0.18	<0.001
At Least One Multifunction Gene (*N* = 115,533)	Linear	0.10	<0.001	0.22	<0.001	0.14	<0.001	0.33	<0.001
	Logarithmic	0.12	<0.001	0.25	<0.001	0.16	<0.001	0.39	<0.001
	Quadratic	0.11	<0.001	0.25	<0.001	0.16	<0.001	0.38	<0.001
	Exponential	0.08	<0.001	0.37	<0.001	0.11	<0.001	0.51	<0.001

Pearson's Correlation Coefficient values (*r^2^*) are reported along with significance values (*p*) for four different types of models for Annotation Count versus Operon Length. Both the Raw and Normalized Annotation Count were modeled with respect to both Unweighted and Weighted Operon Length. The presence of multifunction was also controlled for and the table displays the results for each group (i.e. Single Function versus Multifunction), as well as the results for the combined data.

**Figure 6:**
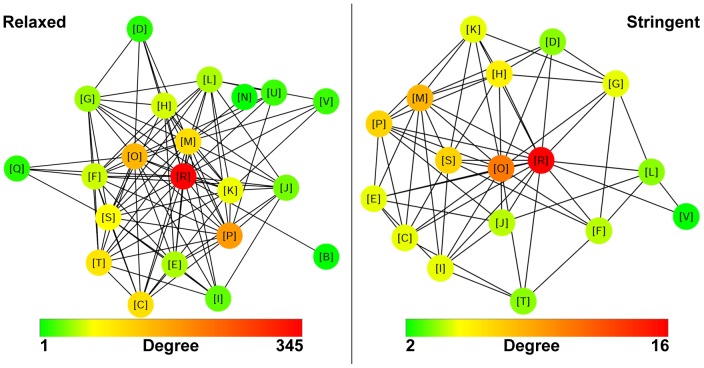
Metagenomic Annotation Networks. Two metagenomic annotation networks were constructed using nine Wastewater metagenomes and the keyword “metal”. The Relaxed network used operons of any length and did not control for multifunction genes while the Stringent network used only operons containing at least three member genes where each member gene was required to be a single function gene. Each node represents a COG category and the node degree is indicated by colour according to the respective legends.

To illustrate how properties such as operon length and the presence of multifunction genes can affect the availability of functional linkages, two metagenomic annotation networks [22] were constructed and subsequently analyzed using Cytoscape 3.0.0 [38]. Specifically the keyword “metal” was used to select target operons from the nine Wastewater metagenomes used in this study (see Methods). The first metagenomic annotation network used relaxed constraints that placed no restrictions on operon lengths or multifunction genes. The relaxed network was derived from 503 operons and contained 21 nodes and 100 edges (see Figure 6). In addition, the normalized annotation count was measured for operons where each member gene had at least one COG and each COG was neither [R], nor [S] (112 operons) and the mean was found to be 0.79 annotations. In contrast, the second metagenomic annotation network was more stringent and required that operons had at least three member genes where each member gene was required to be a single function gene. The stringent network was derived from 112 operons and contained 17 nodes and 64 edges (see Figure 6). The normalized annotation count was measured for operons as described above (18 operons) and the mean was found to be 0.58 annotations.

## Discussion

The results of the gene pair analyses showed only a slight difference between the intergenic distances of genes that shared a functional annotation versus those that did not share a functional annotation. The distributions of gene pair member intergenic distances for Non-matching versus Matching gene pairs were practically indistinguishable with a mean difference of only a few base pairs. As a result, this precluded the establishment of a ‘golden window’ for pairwise gene interactions based on co-directional intergenic distance and this was reflected in the inability to define boundaries for binning gene pairs with respect to match category. Moreover, the success of binning with respect to interaction type reaffirmed that the binning algorithm was well suited for the classification of a dichotomous variable on the basis of intergenic distance, thereby eliminating any concerns about its applicability. Finally, the results of the logistic regression showed that the derivation of a formulaic prediction rule was not achieved using intergenic distance as the only predictor for match category. Overall, this suggests that co-directional gene pairs offer reduced confidence for metagenomic GBA due to difficulty in resolving the existence of functional associations when intergenic distance is the sole predictor of pairwise gene interactions.

In contrast to gene pairs, metagenomic operons, particularly those with substantial lengths, appear to be capable of providing a superior basis for metagenomic GBA. Operon length has been previously used as a metric in operon detection protocols [36], [37]. Here, it was demonstrated that the proportion of distinct annotations within an operon decreases exponentially with increasing operon length. This suggests a progression toward annotative stability indicating that longer operons seem to provide more cohesive functional associations than their shorter counterparts, like interacting gene pairs (i.e. operons of length 2). If we consider a simplified scenario where the probability of two co-directional and proximal genes being falsely predicted as functionally interacting is equal to *p*, then we can see that *P*(∼*O2*) = *p*, where ∼*O2* is a false operon of length 2. If we further assume independence in subsequent pairwise predictions then *P*(∼*O3*) = *p^2^* and in general, *P*(∼*On*) = *p^(n−1)^*, where *n*≥2. Although lacking in empirical elaboration, the previous example demonstrates why it is generally parsimonious to conclude that the probability of predicting an operon by random chance diminishes rapidly with increasing operon length. Such a phenomenon might account for the increased annotative cohesion in longer operons that was observed in the present work because it predicts that shorter operon predictions will collectively contain a greater proportion of spurious operons that will subsequently diminish measurements of annotative cohesion. In other words, while some co-directional and proximal gene pairs represent true operons they exist as an indistinguishable (on the basis of intergenic distance) subset due to the noise caused by random chance occurrences of co-functional annotations in non-operonic gene pairs. Furthermore, the current results indicate that multifunction genes amplify the reduction in annotative cohesion because short-length multifunction containing operons have greater proportions of annotation than their single function counterparts.

The obtained results have a strong bearing on the suitability of using functional associations predicted on the basis of co-directional intergenic distances. For example, broad exploratory networks where fuzziness is desirable might benefit from including operons of any length with no control for multifunction genes. However, the assignment of functional annotation to a gene on the basis of the functions of its operonic co-members requires maximum cohesion thus precluding the use of short-length operons which in turn greatly diminishes the proportion of operons available for such undertakings. Moreover, this type of annotation by GBA must be careful to control for the presence of multifunction genes based on both the current findings as well as other recent work [39]. Interestingly, the metagenomic annotation networks constructed here showed that while selection stringency had an effect on the annotative cohesion of the accepted operons it had only a moderate impact on the number of different nodes and edges in each network, instead greatly affecting the maximum observed node degree. Factors such as the specificity of the keyword and the inherent diversity of the source data are likely to be stronger moderators of network cohesion than operon selection stringency.

The results and interpretation of the present study are predicated on the assumption that it is possible to identify metagenomic operons using the direction and proximity of their member genes. However, such a model [29], [30] is rooted in empirical data from known *Escherichia coli* K12 operons [31] and its extensibility to metagenomic scenarios remains unclear at this juncture. Although it is likely applicable to some taxonomic radius extending from an *Enterobacteriales* centroid, its effectiveness at encompassing broad and diverse taxa is unknown. Given that a primary motivation for using metagenomic data is its accessibility to unculturable and possibly unknown organisms, then it seems of paramount importance to establish a better understanding of the possible configurations for metagenomic operons. One approach might be to examine metagenomic directons to establish correlations between recurring conserved gene groupings and specific taxonomic loci that could provide a clue about alternative operon configurations. An improved ability to recognize metagenomic operons would benefit not only functional annotation efforts but also bioprospecting and other applied and commercial interests. In fact, operon recognition could also provide simultaneous taxonomic indicators by revealing unique polygenic signatures, especially since operon architecture (i.e. gene order) is only weakly conserved across bacterial and archaeal genomes [17], [18].

In addition to potential limitations in recognizing metagenomic operons, the present work used several implementational assumptions that could be addressed by future research. The methods used to determine annotative cohesion considered only the number of different annotation values versus the length of a given operon but not the prevalence of the individual annotations. For example, let us consider two operons, *O_1_* and *O_2_*, each with six member genes, each having one COG, where *O_1_* = {[C], [C], [D], [D], [E], [E]} and *O_2_* = {[C], [C], [C], [C], [D], [E]}. Both operons will have an equal ratio of annotation because they each reduce to the same set of unique elements {[C], [D], [E]}. However, it is clear that *O_2_* is more cohesive than *O_1_* and therefore the metrics used in this work might have reduced the true magnitude of cohesion that occurs in metagenomic operons. Therefore, it is recommended that future appraisals of annotative cohesion consider more sophisticated metrics such as calculations of entropy. In fact, the study of annotative cohesion itself could represent a generally useful pursuit for better understanding the cooperative and co-occurring properties of functional annotations. Similarly, other metrics should be introduced as covariates in an attempt to improve models of annotative cohesion. Repeating this study using alternative annotation hierarchies (e.g. Pfam [40], TIGRFAMs [41], etc.) would also be of potential interest.

Functional associations predicted on the basis of co-directional intergenic distances represent an important homology-free approach for the functional annotation of metagenomic data. Here, evidence for annotative cohesion in metagenomic operons supports the underlying assumption that GBA is indeed applicable to the intergenic distance paradigm. However, depending on the type application caution should be exercised in determining a minimum threshold for operon length, as well as controlling for the potential presence of multifunction genes [39]. Although operonic genes represent only a portion of metagenomic genes, improved operon recognition could increase the utility of metagenomic operons for functional annotation. Moreover, the homology-free nature of intergenic distance permits the assignment of function to genes that do not have corresponding homologs in the various sequence databases. In turn, this would provide a cascading method to expand the breadth of sequence databases beyond their current biases [42], thereby allowing the homology-based annotation of previously unreachable genes. Overall, metagenomic operons offer a largely untapped resource that can drive a variety of annotative and applied interests.

## Supporting Information

Table S1 **Metagenome Properties.** The specific properties of each IMG/M source metagenome are listed with column headers provided in the first row. In addition, the first field (Usage Status) lists the IMG/M identifier for each metagenome in combination with the following colour codes: GREEN = downloaded and used in this study; YELLOW = downloaded but removed due to missing coordinate data; RED = unable to obtain due to file sizes and the timeout policies.
(XLSX)

Dataset S1 **Experimental Data.** The experimental data used in this study are organized as an Excel workbook with genomic gene pairs listed in the first worksheet, metagenomic gene pairs listed in the second worksheet, and metagenomic operons listed in the third worksheet. Column headers are provided in the first row of each respective worksheet.
(XLSB)
